# Accuracy evaluation and addition of improved dihedral parameters for the MMFF94s

**DOI:** 10.1186/s13321-019-0371-6

**Published:** 2019-08-07

**Authors:** Joel Wahl, Joel Freyss, Modest von Korff, Thomas Sander

**Affiliations:** 1 Scientific Computing Drug Discovery, Idorsia Pharmaceuticals Ltd, Hegenheimermattweg 89, 4123 Allschwil, Switzerland; 2 Global Information Systems, Idorsia Pharmaceuticals Ltd, Hegenheimermattweg 91, 4123 Allschwil, Switzerland

**Keywords:** Force field parameterization, Conformer generation, MMFF94s

## Abstract

The Platinum dataset of protein-bound ligand conformations was used to benchmark the ability of the MMFF94s force field to generate bioactive conformations by minimization of randomly generated conformers. Torsion angle parameters that generally caused wrong geometries were reparameterized by conducting dihedral scans using ab initio calculations at the MP2 level. This reparameterization resulted in a systematic improvement of generated conformations.

## Introduction

Mapping molecular structures to energies is of vital importance in computer-aided drug design. Whereas computational quantum chemical methods [such as Density Functional Theory (DFT)] offer high accuracy, their computational cost prohibits their direct application to macromolecules or large databases of molecules, despite the recent increase in computational power [1]. Therefore, molecular force fields still are the method of choice for obtaining potential energies for applications such as molecular dynamics simulations [2, 3], docking [4, 5], or conformational search algorithms [6–8]. In the following article, we will focus on the application of force fields to the generation of low-energy conformers of small molecules and prediction of their potential energies. Considering computer-aided drug design (CADD), all three-dimensional (3D)-based approaches, such as pharmacophore-based screening or docking, are heavily influenced by the quality of the generated small molecule structures. Generating high quality molecular conformers is expected to have a beneficial effect on the predictive power of such methods [9]. Most existing approaches for the generation of conformer ensembles utilize a molecular force field for either conformer refinement or for filtering out high-energy conformations [7, 10–12]. A high quality force field with accurate parameters would thereby improve the outcome of conformational search algorithms [13].

The MMFF94 [14–18] (Merck Molecular Force Field 94) and its static variant for energy minimizations with planar geometries for delocalized trigonal nitrogen centers, MMFF94s [19], emerged as robust and reliable force fields [13, 20, 21] and have been implemented in a multitude of available modeling tools and cheminformatics toolkits [22]. Although the MMFF94s passed the test of time and performs, in general, quite well for the calculation of molecular energies [21], some structural features seem to suffer from sub-optimal parametrization [23]. A multitude of studies [7, 10, 24] benchmarked the ability of different combinations of force fields and conformational search algorithms to regenerate bioactive conformations from protein–ligand-complexes. In our study, the ability of the MMFF94s force field to reproduce bioactive conformations from the recently published Platinum Dataset of bioactive conformations was benchmarked [25]. The Platinum Dataset consists of 2912 protein-bound ligand conformations extracted from the PDB. The chemical space covered by this dataset was shown to be representative of the chemical space of approved drugs. The creators of the dataset furthermore only included ligand conformations that are well supported by the measured electron density, leading to a high-quality set of bioactive conformations. The accuracy of the MMFF94s was compared to an MM2-derived force field [26] which had been implemented in the open-source software DataWarrior [27]. The MMFF94s was also compared to the OPLS3 force field [28] from the Schrödinger Suite [29]. This allowed us, on one hand, to characterize the improvements achieved by the MMFF94s and, on the other hand, to compare its accuracy to a newer, state-of-the-art force field.

It is noteworthy that the bioactive conformation is not necessarily a minimum on the potential energy surface (PES) of a molecule [10, 30]. However, the strain energy for approximately 90% of the bioactive conformations in a set of 150 protein–ligand complexes was estimated to be less than 10 kcal/mol [30]. Therefore, the bioactive conformation usually is located energetically close to a local minimum. Yet for molecules with a rather flat PES, the bioactive conformation can lay far in terms of RMSD (root mean square deviation) from any local or global minimum. On the other hand, approaches that use torsion-angle distributions obtained from the CSD to derive knowledge-based potentials of torsion angles were very successful in reproducing bioactive conformations from the PDB [25, 31]. Moreover, torsion angle distributions obtained from the PDB don’t systematically differ from the ones obtained from the CSD [32]. Therefore, torsion angles in bioactive conformations usually are located in low-energy regions of the torsion-energy profile. Therefore, assessing the ability of a force field to reproduce bioactive conformations using energy minimizations of random conformers has some drawbacks, but in general is still an indicator of the force field’s ability to generate geometries with good quality. Furthermore, it has been shown that approaches that use force field minimization in the conformer generation step are quite successful in reproducing bioactive conformations [13] and can slightly outperform approaches based on statistically derived torsion potentials [31]. If the force field minimization would be a badly chosen strategy for the generation of bioactive conformations, such results would not have been obtained.

In our approach, torsion angles were detected that systematically differed between the bioactive conformation obtained from crystal structures and the closest conformer obtained by minimization with the MMFF94s. This was facilitated by assigning every rotatable bond a unique identifier (TorsionID) as a canonical description of the local environment, similar to the concept of the Torsional Fingerprints [33] (more information follows in “Materials and methods” section). PES (Potential Energy Surface) scans were conducted for this set of problematic dihedral angles employing ab initio calculations at the MP2 level using model compounds. Although conformational energies of force fields do not solely depend on the torsional parameters, systematic addition or reparameterization of torsional parameters may improve both geometry prediction and conformational energy calculations [28, 34]. The advantage of this approach is that one can specifically improve the accuracy of the force field for specific substructures without altering predictions for the rest of the chemical compound space. We compared the torsional profiles obtained by MMFF94s with the ones from the ab initio calculations and reparameterized the corresponding torsion parameters.

## Materials and methods

### Test set for conformer generation

The Platinum Diverse Dataset [25] 2017_01 was used.

### Generation and processing of random conformers

For every molecule in the test set, up to 1000 different conformers were generated by random assignment of dihedral angles in 30° steps. Meaning that all rotational bonds could take values from 0° to 360° in 30° steps. Ring conformations were not altered by this protocol. The randomly generated conformers were optimized (300 steps conjugate gradient, 5000 steps LBFGS (limited-memory Broyden–Fletcher–Goldfarb–Shanno) by means of the MMFF94s, MM2 [26] (both implemented in-house), and OPLS3 [28, 35] force field. The calculations were carried out in gas-phase with a dielectric constant of 4. The estimated dielectric constant for proteins ranges between 4 and 20 [36, 37] and depends on the composition of the protein and the location of the binding site. The chosen value for a conformational search is to a certain degree arbitrary and cannot represent all states equally well, but a value of 4 in our experience usually yields good results for conformational searches using force fields.

Subsequently, all optimized conformers lying within an energy window of 10.0 kcal/mol compared to the conformer with the lowest energy were categorized as “low energy conformers”. The root mean square deviation (RMSD) of each of these conformers to the crystal structure was then calculated and the lowest value of each force field for each molecule was taken to compare the predictive power of the force fields.

### TorsionID

**Fig. 1:**
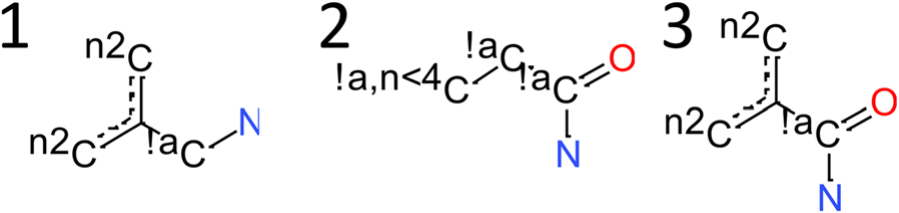
Example representations of torsion fragments. Example 1 refers to the dihedral angle of the rotation around a C–C bond, whereas C_1_ is part of a delocalized system, neighboring two C-atoms with two neighbors each (^n2^C). C_2_ is a non-aromatic C (^!a^C) bound to a nitrogen atom. In example 2, we again refer to the rotation around a C_1_–C_2_ bond. C_1_ is a non-aromatic carbon, bound to a non-aromatic carbon with less than 4 neighboring atoms (C^!a,n<4^; sp^2^ or sp hybridized). C_2_ is an amide carbonyl carbon. Example 3 is a combination of Examples 1 and 2, where C_1_ is part of a delocalized system, neighboring two C^n2^ atoms, and C_2_ is an amide carbonyl carbon

In order to identify systematic errors in the MMFF94s caused by inaccurate or missing parameters, we searched for types of dihedral angles that were often predicted incorrectly. In order to achieve this, the concept of TorsionIDs was used. The TorsionID is a string representation of a torsion angle, that unambiguously identifies the rotatable bond and its vicinity, containing information about the chemical environment (aromaticity, hybridization) of the atoms defining the torsion angle, while still keeping a certain level of generality in order to conduct statistical analysis of similar torsion angles and forming torsional libraries. It furthermore encodes information about stereochemistry and symmetry. TorsionIDs can be assigned to bonds that are single bonds, non-aromatic bonds and not member of a ring with ≤ 5 members. The string representation can be converted back into a molecular fragment and visualized (examples are given in Fig. 1).

The algorithm constructs a molecular fragment that uniquely describes the relevant chemical properties of the torsion situation. The fragment usually contains the atoms that form the central bonds and the atoms directly connected to them. If the bond is though part of a consecutive sp–sp atom chain, then all linear atoms are part of the fragment together with the two end atoms. The TorsionID encodes for every atom of the fragment its element, aromaticity, number of neighbours and in addition flags sp2 hybridized nitrogens specially. For the bonds, the order is encoded and delocalized bonds are flagged specially. Aromaticity is determined for isolated ring systems with a maximum ring size of 7 and in principal uses Hückel’s rule. Delocalized bonds have different bond orders in mesomeric structures that are regarded as energetically equivalent, such as in 6-membered aromatic rings. Rings in 5-memberd heterocyclic structures on the other hand are not regarded as being delocalized. An sp2 flag is assigned to nitrogens that are aromatic, part of an amide, enamine or in resonance with an aromatic ring. Nitrogens with ortho substituents that prevent planarity do not receive the sp2 flag.

For every molecule in the test set, we compared the torsion angles of all rotatable bonds of the bioactive conformation with the ones of the closest low-energy conformer from the force field minimizations. If the deviation was larger than 30°, the TorsionID of this dihedral was determined and the cumulative number of wrong predictions for this TorsionID was increased. To take into account molecular symmetry, every TorsionID had a symmetry element assigned to it that was used to calculate the symmetry-corrected deviation. The symmetry of the two atoms defining the rotatable bond of the torsion angle was determined (C1, D1, D2, or D3 symmetry group), whereas the D3 symmetry was treated equally to D1 for simplicity. This gives rise to the following combinations: C1C1, C1D1, C1D2, D1D1, D1D2, and D2D2. A mapping function f($\varphi$) was then applied to get the symmetry-corrected value of the calculated deviation $\varphi$, where $\varphi$ can take values from − 180˚ to + 180˚.

$${\text{f}}_{C1C1} \left( \varphi \right) = {\text{f}}_{C1D1} = \left\{ {\begin{array}{ll} {\varphi + 360} & \quad {\varphi < 0} \\ \varphi & \quad{\varphi \ge 0} \\ \end{array} } \right.$$

$${\text{f}}_{C1D2} \left( \varphi \right) = \left\{ {\begin{array}{ll} {\varphi + 180} & \quad {\varphi < 0} \\ \varphi & \quad {\varphi \ge 0} \\ \end{array} } \right.$$

$${\text{f}}_{D1D1} \left( \varphi \right) = \left| \varphi \right|$$

$${\text{f}}_{D1D2} \left( \varphi \right) = {\text{f}}_{D2D2} \left( \varphi \right) = \left\{ {\begin{array}{ll} {\varphi + 180} & \quad {\varphi < 0} \\ {180 - \varphi } & \quad {\varphi > 90} \\ \end{array} } \right.$$

### Torsion scans

Torsion scans with the original MMFF94s were conducted using the “Relaxed Coordinate Scan” module integrated in the MacroModel [38] program from the Schrödinger suite. Scans at the ab initio level were conducted using Jaguar [39, 40] at the LMP2/6-31G* level. Single point energies of the minimized structures from the scan were calculated by GAMESS-UK [41] at the MP2/cc-pVTZ level, this strategy was chosen to be as consistent as possible with the parametrization procedure of the original force field [18]. In the original paper, torsion profiles were calculated from geometries optimized at the MP2/6-31G* level and the single point energies were calculated at the MP2/TZP (triple zeta with polarization) level, with a custom defined basis set.

### Reparameterization of torsion parameters

Torsional energies are described by Eq. 1 for the MMFF94s [18]:

1 $$ET_{ijkl} = 0.5\left( {V_{1} \left( {1 + cos\varphi } \right) + V_{2} \left( {1 - cos2\varphi } \right) + V_{3} \left( {1 + cos3\varphi } \right)} \right)$$

The torsional energy is expressed as a sum of a onefold term with barrier height V_1_, a twofold term with barrier height V_2_ and a three-fold term with barrier height V_3_. Every atom quadruple i-j-k-l has a triplet of parameters associated with it. In case there is no parameter for an atom quartet, the MMFF94s uses a set of wildcard parameters or parameters of a chemically related quadruple [18].

We employed two strategies for the reparameterization of the torsion parameters, a systematic strategy and a strategy based on a genetic algorithm. In the systematic strategy, the torsion parameters related to a specific series of atoms *i*-*j*-*k*-*l* were systematically changed (step size: 0.1) and the merit function was evaluated. The merit function consisted of the (RMSD) between the PES obtained by the force field compared with the MP2/cc-pVTZ curve. The systematic strategy was applied for cases where only the torsion parameters for one atom quadruple *i*-*j*-*k*-*l* needed to be reparameterized, as for the phenyl-pyrroles (see “Results” section). The GA strategy was applied for the *N*-aryl amides containing 5-membered heterocycles. More details are given in the result section.

## Results

### General accuracy of the MMFF94

Using the Platinum Diverse Dataset 2017_01, a conformation with a similarity of 0.5 Å or better to the bioactive conformation could be identified from the low-energy conformational pool of the MMFF94s for 53% of the cases, compared to 43% achieved by the MM2. This trend continued for higher RMSD thresholds, with 89% of the bioactive conformers reproduced with an RMSD of 1.0 Å or better for the MMFF94s, compared to 81% for the MM2. Therefore, compared to the MM2 force field, the s had a much higher likelihood to generate low-energy conformers with high similarity to the bioactive conformation.

Furthermore, the MM2 failed to process 278 of the 2859 structures of the dataset, compared to 26 unprocessed structures for the MMFF94s. Hence the MMFF94s covers the chemical space considerably better than the MM2 (Table 1).

**Table 1:** Summed up ratio of bioactive molecules recovered with varying RMSD thresholds for different force fields

RMSD	Platinum Diverse Dataset 2017_01 (2581 molecules)		
	MMFF94s	OPLS3	MM2
< 0.5 Å	0.53	0.55	0.43
< 1.0 Å	0.89	0.90	0.81
< 1.5 Å	0.98	0.97	0.92
< 2.0 Å	0.99	0.99	0.96
Failure rate (%)	0.8	0.8	9.7

Note that the original Platinum Diverse Dataset 2017_01 consists of 2859 molecules. The 2581 molecules correspond to the subset that could successfully be processed by all three force fields. The failure rate indicates the percentage of the structure of the total dataset that could not be processed by the force field

These results confirm that adding the MMFF94s force field to DataWarrior improves the accuracy of the small-molecule structure generation. The recovery rates of MMFF94s are similar to the recently published OPLS3 force field that employs many more parameters than the MMFF94s and leads to a more accurate prediction of conformational energies [28]. The MMFF94s is a reliable choice for conformational search procedures employing force field minimizations, where correct location of the minima is usually of higher importance than correct reproduction of the barrier heights for interconverting the conformers. The latter, however, is of crucial importance for Molecular Dynamics or Monte Carlo Simulations, two applications that are not covered in our study.

### Reparameterization of problematic substructures

**Fig. 2:**
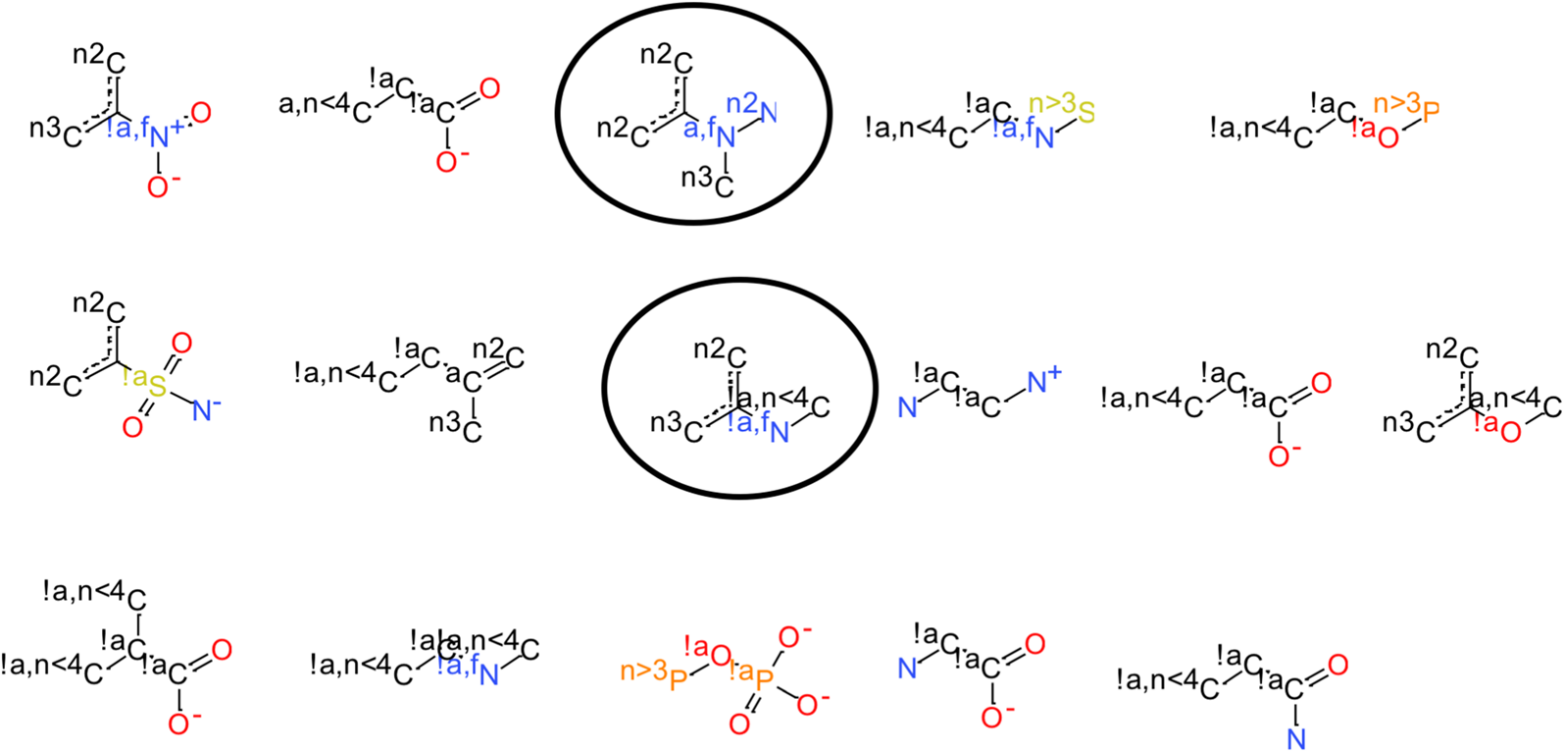
Torsional fragments for which the MMFF94s-minimized structures exhibited systematic deviation of the central dihedral angle compared to the experimental structures

Whereas our strategy to identify problematic substructures revealed a multitude of different examples (Fig. 2), two compound families emerged as being systematically incorrectly parameterized: *N*-aryl amides and phenylpyrroles (circles in Fig. 2). Due to their pharmacological relevance and high occurrence in drug-like structures, reparameterization of the related torsion parameters was expected to result in an overall improvement of theMMFF94s' usefulness for applications in computer-aided drug design projects. In Fig. 2, several other substructures with systematic deviation of torsion angles generated from force field minimization compared to the torsion distributions from experimental structures are shown. Some of these dihedral angles describe the rotation of terminal group, where a reparameterization is not expected to vastly affect the outcome of a conformational search algorithm. Furthermore, some of the dihedral angles are associated with low rotational barriers. In this case, deviations are energetically possible and the conformational strain can be compensated by favorable intermolecular or intramolecular interactions. In this study, we focused on the reparameterization of the two aforementioned compound families.

#### *N*-aryl amides

The MMFF94s force field vastly overestimates the rotation barrier around the C_arom_–N_amide_ bond for aryl amides. This finding is in agreement with a recent study that compared torsional profiles of various fragments obtained by different force fields and ab initio methods [24]. As a result, the MMFF94 has a tendency to force aryl amides into a planar orientation, even in case of double *ortho*-substitution.

**Fig. 3:**
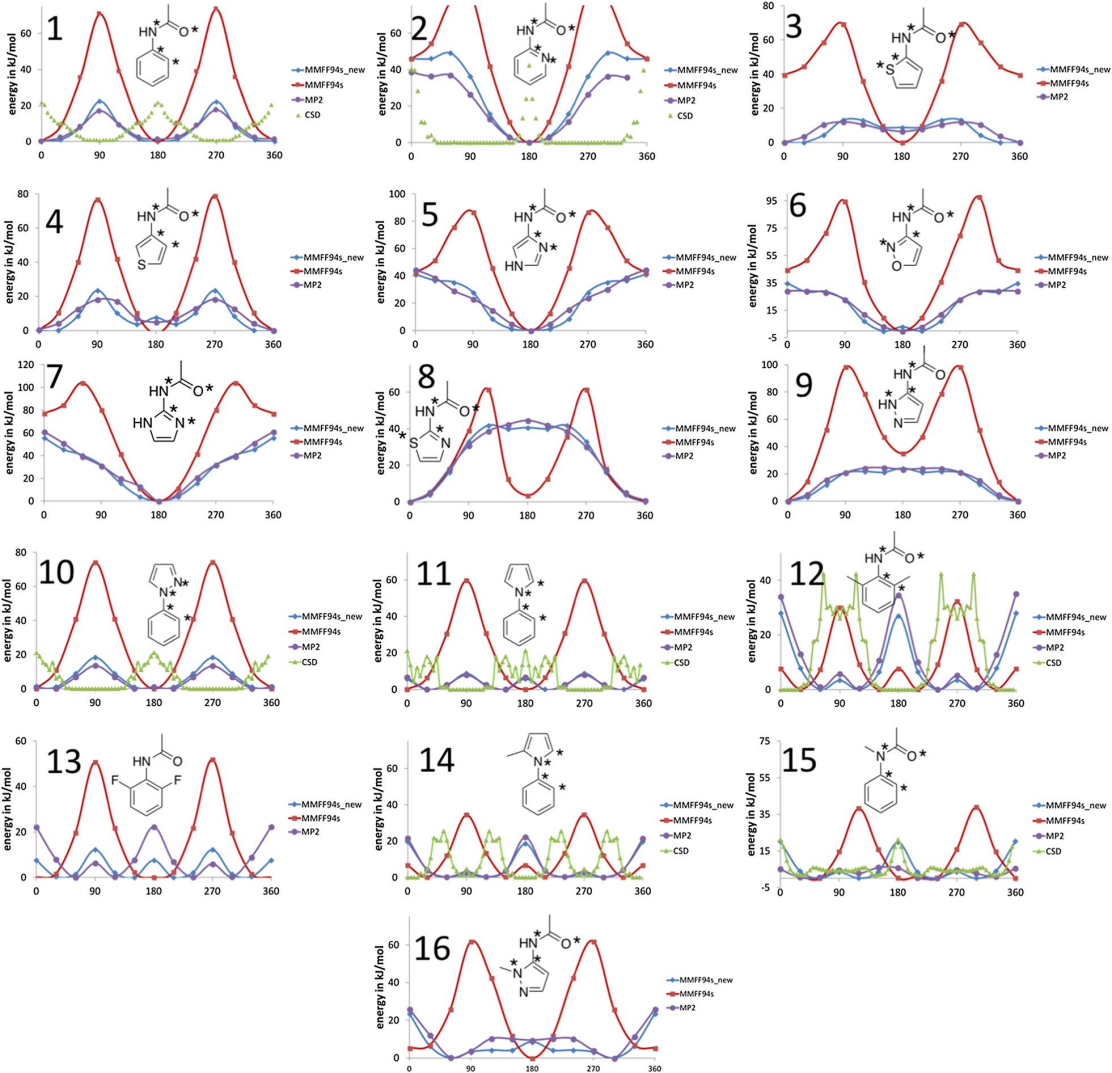
Torsional energy profiles for various substructures with problematic torsion angles for the MMFF94s. The four atoms defining the scanned torsion angles are indicated by asterisks. Conformational energies are given in kJ/mol. These structures are not present as ligands in the platinum dataset. Torsion histograms from the CSD with relative frequencies were added for several compounds

We evaluated a range of aryl amides with different aromatic systems, such as phenyl, pyridyl and 5-membered heterocyclic aromatic structures (compounds **1**–**9**, Fig. 3). The plots indicate that all systems suffer from an overestimation of the rotational barrier. We first derived new parameters for the *N*-phenyl amide and checked the transferability towards the remaining aryl-amide systems. The reparameterization for *N*-phenyl amide (Fig. 3, compound **1**) was straightforward. In principle, rotation around the C_arom_–N_amide_ bond can be described by two torsion quadruples: (1) C_arom_–C_arom_–N_amide_–C_amide_ or (2) C_arom_–C_arom_–N_amide_–H_amide,_ corresponding to MMFF94 atom types 37-37-10-3 and 37-37-10-28 respectively. In the original implementation of the MMFF94, the torsional parameters for the quadruple 0-10-37-0 were defined, where 0 corresponds to an atomic wild card. The corresponding parameters are V_1_ = 0.0, V_2_ = 6.0, V_3_ = 0.0. Our systematic search strategy suggested that lowering V_2_ to 2.7 gives the best agreement with the MP2 PES.

Considering force field parameterization, a balance between accuracy and generality has to be found. Usage of wild-card parameters improves the generality and leads to fewer parameters but may result in decreased accuracy. We tested the transferability of our parameters by conducting PES-scans for the rotation around the C_arom_–N_amide_ for *N*-(2-pyridyl) amide (Fig. 3, compound **2**) and the new parameters results in a good agreement of the torsion potential with the MP2 results, showing that the approach with the wild-card parameters leads to sufficient accuracy for *N*-phenyl amides. Next, we examined if the parameters from the *N*-phenyl amides can be transferred to *N*-aryl amides containing 5-membered heterocycles. In general, this resulted in an unsatisfying reproduction of the QM-potentials (results not shown), leading to the conclusion that these subsystems need specific parameterization.

The challenge associated with the parameterization of these systems is the breakage of the ring-symmetry due to substitution at the one of the ring carbons. MMFF94s has two different atom types for the carbons in 5-membered heteroaromatic compounds: atom type 63 (alpha carbon) and atom type 64 (beta carbon). The original MMFF94s possesses torsional parameters for the atom quadruples 0-63-10-0 and 0-64-10-0. Yet, refining these parameters using a systematic search did not result in a satisfactory outcome and showed that reproducing the torsional preferences and barriers for the *N*-aryl amides needs more specific parameters. We derived parameters for the atom quadruples in Table 2 using a subset of structures containing the necessary torsional angles. These structures (compounds **1**–**9**, Fig. 3) compose our parametrization set that we used to fit the torsional parameters for the corresponding atom quadruples. This added redundancy for some of the atom quadruples since they occur in more than one structure. The refitting of the parameters was conducted by a genetic algorithm with the summed up RMSDs of all input molecules as the fitness function, an established strategy in force field parametrization [42]. For most torsion quadruples, only V2 was optimized whereas V1 and V3 were set to zero. V1 was optimized when the correct energy difference between a *cis* and *trans*-orientation could not be reproduced (see Eq. 1).

**Table 2:** Newly derived torsion parameters obtained by fitting the torsional profiles to the PES obtained from torsion scans conducted at the MP2 level

TT-I-J-K-L	A-B-C-D	V1, V2, V3
0-0-10-37-0	*-(NC=O)-CB-*	0.0, 2.7, 0.0
0-3-10-64-66	(C=ON)-(NC=O)-C5B-N5B	− 0.31, − 5.82, 0.0
0-28-10-64-66	(HNCO)-(NC=O)-C5B-N5B	0.00, 1.29, 0.00
0-3-10-64-63	(C=ON)-(NC=O)-C5B-C5A	0.00, 7.97, 0.00
0-28-10-64-63	(HNCO)-(NC=O)-C5B-C5A	0.00, 7.97, 0.00
0-3-10-64-64	(C=ON)-(NC=O)-C5B-C5B	2.47, − 3.26, 0.00
0-28-10-64-64	(HNCO)-(NC=O)-C5B-C5B	0.00, 4.76, 0.00
0-3-10-64-65	(C=ON)-(NC=O)-C5B-N5A	− 0.76, 8.14, 0.00
0-28-10-64-65	(HNCO)-(NC=O)-C5B-N5A	0.00, 5.27, 0.00
0-44-63-10-3	STHI-C5A)-(NC=O)-(C=ON)	− 11.5, 6.60, 0.00
0-44-63-10-28	STHI-C5A)-(NC=O)-(HNCO)	0.00, 3.34, 0.00

Atom types: 0 = wild card, 3 = carbonyl carbon, 10 = amide nitrogen, 28 = amide hydrogen, 37 = aromatic carbon, 44 = thiophene sulfur, 63 = alpha carbon, 64 = beta carbon, 65 = alpha aromatic heterocyclic 5-ring nitrogen, 66 = beta aromatic heterocyclic 5-ring nitrogen. TT corresponds to the torsion type index [18]

Comparing the fit of the force field potentials to the MP2-PES (Fig. 3) shows a systematic improvement due to the reparameterization, leading to a much more realistic representation of the torsion barriers and conformational preferences. Noteworthy examples are the amidated thiophenes. The *N*-(2-thiophene)amides (Fig. 3, compounds **3** and **8**) have a preference for an S–C–N–C angle of 0°, due to a S–O chalcogen interaction [43]. Since the simple point charge models of traditional force fields such as the MMFF94s cannot account for the anisotropic nature of the charge distribution in sulfur atoms, the conformational preference for this subclass of compounds has to be corrected by the addition of a specific V1 torsion parameter for the quadruple 44-63-10-3 that stabilizes an angle of 0°. In our case, the new torsion parameters lead to a much better description of the PES of these amidated thiophenes.

Yet, assigning specific torsion parameters to capture the effects of a non-standard 1–5 interaction (in this case, thiophene sulfur with amide oxygen) bears a risk of overfitting. Addition of these torsion parameters leads to an improved description of the PES of the *trans*-amide. For the PES of the *cis*-amide, on the other hand, the S–O interaction is not feasible since the amide oxygen is pointing away from the sulfur. In this case, the in-plane conformation is artificially overstabilized by these specific parameters. However, we checked the CSD for occurrences of example 3 with a *cis*-amide bond and could not detect any. We conclude that in general for this subclass of compounds, the *trans* isomer is vastly preferred, and therefore, our approach is a valid solution leading to correct prediction of the conformational preference of compound **3**.

#### Phenylpyrroles

Compounds **10** and **11** (Fig. 3) were used for the reparameterization of the torsional parameters describing the rotation around the central bond connecting the two aromatic rings. As for the aryl amides, the MMFF94s suffers from a vast overestimation of the rotational barrier. In the case of 1-phenylpyrazole (Fig. 3, compound **11**), this leads to a wrong location of the energetic minimum. Whereas MMFF94s predicts full planarity for the biaryl system, the results from MP2 indicate that a slight out-of-plane conformation is energetically favorable. The PES obtained from MP2 is flat with rotational barriers of about only 2 kcal/mol. In case for 1-phenylpyrrole (Fig. 3, compound **10**), the MMFF94s correctly predicts the planarity of the system, but again penalizes out-of-plane conformations to an overproportional degree. These shortcomings of the MMFF94s are overcome by correcting the torsional parameter for the rotation about the C–N bond. The original MMFF94 has parameters for the quadruple 0-37-39-0. We refined V2 with the systematic search and got satisfactory results. The correct conformational preference for the 1-phenylpyrrole is now reproduced and for both compounds, the rotational barriers are in much better agreement with the ab initio data (Table 3).

**Table 3:** Newly derived torsional parameters for phenylpyrroles

TT-I-J-K-L	V1, V2, V3
1-0-37-39-0	0.0, 2.6, 0.0

### Transferability of new parameters

Deriving general torsional parameters from simple model compounds containing the atom quadruple of interest is an established strategy in the development of additive force fields [44, 45]. Ideally, these parameters also reproduce conformational energies in the presence of different substituents. We performed dihedral scans for *N*-aryl amides (compounds **12, 13**, **15**, **16**, Fig. 3) and a phenylpyrrole (compound **14**, Fig. 3) with differing substituents and checked if the PES is still reproduced well.

We can conclude that for methylation at an aromatic carbon in *ortho*-position (Fig. 3, compounds **12**, **16**) to the amide group, the parameters are still valid and display good transferability. For compound **12**, the original MMFF94 locates the minimum quite close to the in-plane conformation and only gives a small conformational strain for a dihedral angle of 0° or 180°. With the new parameters, the PES from the MP2 calculation is perfectly reproduced with correct location of the minima and correct barrier heights. The same applies to compound **14**, where the new parameters perfectly reproduce the MP2-PES. For compound **16**, transferability of the parameters is still satisfying with correct location of the minima, but overall the energy profile deviates slightly from the MP2 result.

In case of *ortho*-fluorination (compound **13**), the results are less promising. Whereas the new parameters certainly are a large improvement compared to the original implementation, the energetical strain for the in-plane conformation (0° and 180°) is overestimated by a mere 10 kJ/mol. If the substitution has only a steric or electrostatic effect on the conformational energy of the molecule, the transferability of the torsional parameters is not expected to be problematic, assuming that the nonbonded interactions are treated with sufficient accuracy. However, if the substituent influences the degree of delocalization in a system, influences hyperconjugation, or leads to an interaction not covered by classical force fields, the transferability of the torsional parameter is limited. Here, the presence of the fluorine probably influences the degree of delocalization in the system. Also for compound **15**, the MP2-PES is not reproduced well. One challenge associated with N-methylation for phenylacetamides is the fact that the MP2 calculations yield non-planar amide nitrogen atoms for these compounds and therefore the PES is non-symmetric. Apart from this, the in-plane conformation is penalized by a too high degree with the new parameters. One possible explanation is that the N-methylation leads to a stronger orbital overlap of the nitrogen nonbonded p-orbital with the π-system of the ring. This stronger delocalization stabilizes the planar conformation.

In case of compound **15**, since the methyl-carbon is a member of the atom quadruple defining the torsion angle of interest, a new parameter 0-3-10-37-0 can be derived, with 3 being the atom type for aliphatic carbons. For compound **13**, however, the fluorine is not part of the atom quadruple since both non-fluorinated and fluorinated aromatic carbon possess the same atom type (37), the effect of the fluorination cannot be taken into account. One possible solution would be to add a new parameter for fluorinated aromatic carbons. However, this would also lead to additional parameters (Lennard–Jones, Partial Charges, Bond-Stretching, Angle-Bending) that need to be derived for this new atom type.

### Impact of the new parameters on conformational searches

We assessed how the new parameters influence the ability of a conformational search algorithm based on force field minimizations to reproduce bioactive conformers taken from the PDB (Protein Data Bank). We applied the strategy described in “Materials and methods” section and took a subset from the platinum dataset with compounds that contain at least one of the reparameterized torsion angles (Table 4).

**Table 4:** Summed up ratios of bioactive molecules recovered with different RMSD values

RMSD	*N*-aryl amides (272 molecules)			Phenylpyrroles (63 molecules)		
	MMFF94s	MMFF94s new	OPLS3	MMFF94s	MMFF94s new	OPLS3
< 0.5 Å	0.34	0.43	0.41	0.52	0.59	0.52
< 1.0 Å	0.85	0.89	0.89	0.87	0.90	0.83
< 1.5 Å	0.98	0.98	0.98	1.00	0.97	0.98
< 2.0 Å	0.99	0.98	0.99	1.00	0.98	1.00

For both subsets, the new parameters (MMFF94s_new) provided a significant improvement over the original implementation of the MMFF94s. In case of the *N*-aryl amides, the share of bioactive conformers that were reproduced with an RMSD of smaller than 0.5 Å increased by 9% (0.43 vs. 0.34). Looking at a recovery of 1.0 Å or better, there was a 4% improvement with the new parameters and even a slightly better recovery rate than OPLS3. The same applied to the set with the phenylpyrroles, where for a recovery of 0.5 Å or better, the new parameters lead to a 7% improvement and to a 3% improvement for 1.0 Å. Again, the MMFF94s_new performed even better than the OPLS3 force field.

**Fig. 4:**
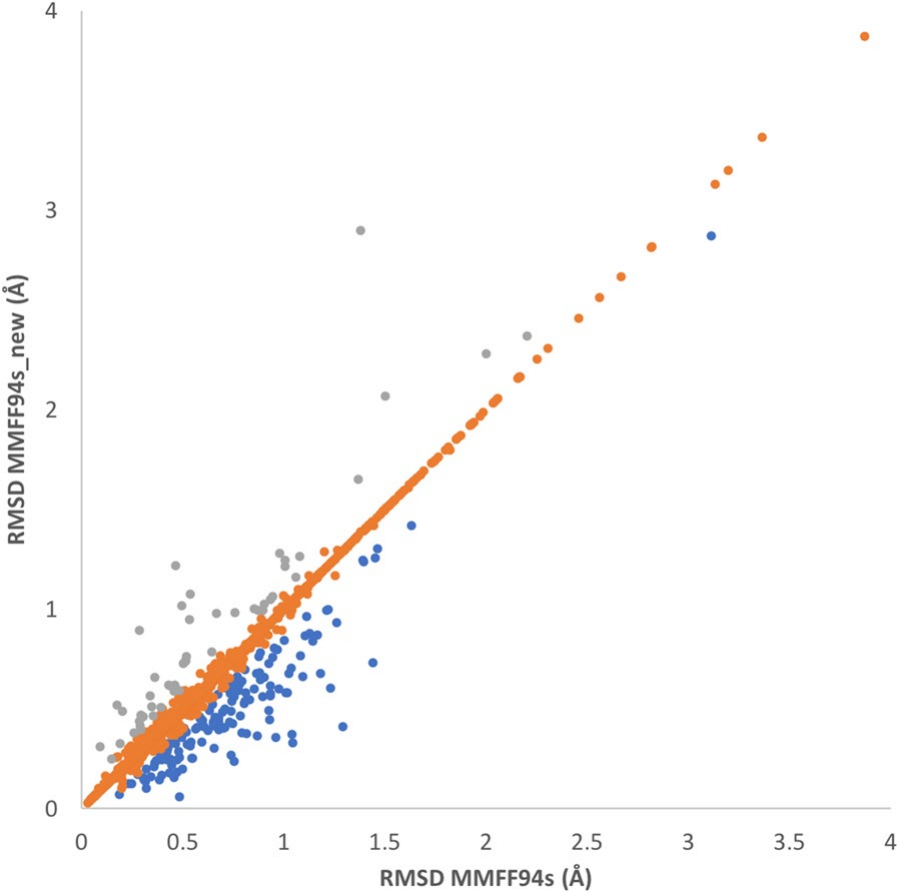
Plot of RMSDs between the closest low-energy conformer and the bioactive conformation for the original MMFF94s against the MMFF94s with the new parameters (MMFF94s_new) for 2833 structures from the Platinum Diverse Dataset. Data points where the unsigned difference between the two RMSD values is less than 0.1 are colored orange, data points where the MMFF94s_new performs better are shown in blue, whereas the grey data points indicate a better performance by the original MMFF94s

A comparison of the RMSD values with respect to the bioactive conformations for the original MMFF94s and the reparameterized force field is depicted in Fig. 4 for the whole Platinum diverse dataset. For 146 structures, the MMFF94s_new showed a better recovery rate in comparison with the MMFF94s, whereas in 47 cases, the MMFF94s performed better.

## Conclusion

The MMFF94s is a built-in functionality of the DataWarrior program. Its ability to locate bioactive conformers among a force-field minimized pool of low-energy conformers was compared with the ability of the MM2-derived Actelion force field (also implemented in DataWarrior) and the modern OPLS3 force field. It was shown that the MMFF94s provides an improvement in terms of robustness and accuracy over the MM2-derived force field. Furthermore, it performs comparably well as the OPLS3 force field for the described application.

Still, the MMFF94s suffered from insufficient parameterization for *N*-aryl amides and phenylpyrroles. Ab initio PES scans with single point energies at the MP2 level were conducted and a set of new torsional parameters was fitted that reproduce the torsional energy profiles from the MP2 scans. The newly added parameters lead to an improved recovery rate of bioactive conformers for the substructures of interest.

Regarding future improvements of the force field, the addition of new atom types would be required to improve the accuracy. The drawback of adding new atom types is that it is associated with considerable work for parametrization, since also valid bond-stretching, angle-bending, bond charge increments, and Lennard-Jones parameters have to be derived.

Optimization of torsional parameters can lead to an improvement of the force field accuracy for certain substructures, but for a more systematic improvement, a better description of the electron distribution of the molecule would also be beneficial. One possibility is to derive improved partial charge models [46]. The next step would be to more properly account for charge anisotropy (off-site charges [47, 48] or atomic multipoles [49, 50]). In this case, special interactions such as the intramolecular S–O chalcogen bond would not have to be covered by specific torsion parameters and, in general, the force field would yield more accurate conformational energies with less torsional parameters needed.

The new parameters are available in DataWarrior, where the user can choose between the original implementation of the MMFF94s and the implementation with the altered parameters.

## Data Availability

The platinum dataset can be downloaded from http://www.zbh.unihamburg.de/platinum_dataset. DataWarrior can be downloaded from http://www.openmolecules.org/dataWarrior/download.html.

## Abbreviations

CADD: computer-aided drug design
PDB: protein database
RMSD: root mean square deviation
DFT: density functional theory
PES: potential energy surface
MP2: second order Møller–Plesset perturbation theory
LMP2: local MP2
QM: quantum mechanics
LBFGS: limited-memory Broyden–Fletcher–Goldfarb–Shanno
